# THE USE OF SELF-MANAGEMENT TECHNOLOGY AND HEALTH CARE UTILIZATION IN LATER LIFE

**DOI:** 10.1093/geroni/igz038.2548

**Published:** 2019-11-08

**Authors:** Darren Liu, Takashi Yamashita, Betty Burston, Jennifer Keene

**Affiliations:** 1 Des Moines University, Des Moines, Iowa, United States; 2 University of Maryland, Baltimore County, Baltimore, Maryland, United States; 3 University of Nevada Las Vegas, Las Vegas, Nevada, United States

## Abstract

Health-related websites and applications are useful tools for self-management in health care settings. However, the limited accessibility due to the lack of technology-related skills can be a “digital divide” issue that particularly affects older adults. To better understand the role of such tools in later life, this study examined the use of internet-based technology in relation to health care service utilization. A nationally representative samples of adults age 55 years and older (n = 1,157) were obtained from the health behaviors module of the 2014 Health and Retirement Study (HRS) in the U.S. We analyzed the use of health management websites and apps and its association with health care service utilization measured by the number of doctor visits during the last two years before the interviews. Negative binomial regression was used to model the number of doctor visits as a function of the health management technology use and other covariates. Results revealed that the level of technology use (b = 0.41, p < 0.05) was significantly associated with a 50% greater number of doctor visits. Such technology use was significantly more common among adults with good health than those with poorer health outcomes (17.32% & 9.60%, respectively, p < 0.05). The findings suggest that healthy adults take advantage of available internet-based self-management tools and proactively utilize health care services more often than individuals who can be categorized as “unhealthy”. There is a continuing need for research on introducing strategies that can ultimately reduce health disparities by decreasing the digital divide.

